# Dose Reduction/Discontinuation of Antipsychotic Drugs in Psychosis; Effect on Cognition and Functional Outcomes

**DOI:** 10.3389/fpsyt.2018.00447

**Published:** 2018-09-20

**Authors:** Yoshie Omachi, Tomiki Sumiyoshi

**Affiliations:** ^1^Department of Psychiatry, National Center Hospital, National Center of Neurology and Psychiatry, Tokyo, Japan; ^2^Department of Preventive Intervention for Psychiatric Disorders, National Institute of Mental Health, National Center of Neurology and Psychiatry, Tokyo, Japan

**Keywords:** first episode psychosis, schizophrenia, discontinuation, maintenance, antipsychotics, cognition, social function, functional outcome

## Abstract

**Backgrounds:** There is a debate regarding the optimal timing of discontinuation of antipsychotic drugs in patients with first episode psychosis (FEP) or schizophrenia. We aimed to provide a review of the literature on which strategy (medication maintenance vs. dose reduction/discontinuation) is more likely to maximize outcomes, such as cognition and social function.

**Methods:** Using PubMed, the Cochrane Library and systematic reviews, articles published between 2007 and 2018 were reviewed, which investigated the effect of dose reduction/discontinuation vs. maintenance treatment on measures of cognition and/or social function in FEP and schizophrenia.

**Results:** Six studies were identified; 2 studies reported on cognition while 4 studies concern social function. All studies except one reported that improvement of functional outcomes in remitted patients with FEP or schizophrenia allocated to a dose reduction/discontinuation arm was equal to or better than that in patients for whom medication doses were maintained. One trial of social function with a 1-year follow-up period found a greater improvement in the medication maintenance group, while no group difference was observed with 3-year and 10-year follow-up periods. On the other hand, a 7-year follow-up study found a superiority for the dose reduction/discontinuation regimen in terms of social outcome. Two studies on cognition with a short follow-up period reported a greater improvement for the dose reduction/discontinuation group.

**Conclusions:** Information on cognition and social function has been relatively sparse. These measures of functional outcome should be considered in deciding which strategy of antipsychotic treatments is beneficial in individual cases with FEP or schizophrenia.

## Introduction

There is controversy about the continued use of antipsychotic drugs in patients with psychotic disorders, including schizophrenia. For example, treatment guidelines for first episode psychosis (FEP) recommend at least 1-year of antipsychotic treatment following remission (1). Antipsychotic medication may affect relapse and remission rates over time. In fact, randomized controlled trials (RCTs) have reported a considerably high relapse rate after dose reduction/discontinuation of antipsychotic treatment (2–5). From the clinical point of view, the maintenance/discontinuation debate should also encompass other aspects, such as functional outcome.

Functional outcome consists of several domains, such as psychosocial skill acquisition, instrument skills/social problem-solving ability, and community outcome/daily activities (6). A general consensus is that recovery in psychosis contains achievements of a personally acceptable quality of life and feeling of self-esteem (7). These outcome measures have been suggested to receive a higher priority than symptom management for young people with psychosis (8). Specifically, there is convincing evidence for an association between cognition and functional recovery in schizophrenia, as cognition may provide a better correlate of functional outcome than psychotic symptoms (6, 9). Accordingly, Sumiyoshi et al. (10) reported temporal associations between cognition and social function in patients with schizophrenia. Longitudinally, Fu et al. (11) found that performance on tests of attention, verbal learning, and verbal working memory are associated with social function throughout a 4-year observation period. Consequently, efforts have been made to develop therapeutics for disturbances of cognition (10).

To date, effects of antipsychotic drugs on cognition in patients with schizophrenia have been intensively examined. Initially, the second-generation antipsychotic drugs (SGAs) were suggested to ameliorate cognitive impairment more effectively than the first-generation antipsychotic drugs (FGAs). However, results from recent meta-analyses of RCTs on the effect of SGAs vs. FGAs, or SGAs vs. placebo indicate that only clozapine elicits cognitive benefits in patients with schizophrenia (12–14). So far, no consensus has been established as to an appropriate duration of antipsychotic treatments, particularly with regard to cognitive and social outcomes.

Some clinicians may feel that the quality of life (QOL) in patients with FEP would be better if antipsychotic medications are discontinued following remission (15). To our knowledge, there has been little information on the effects of maintenance vs. dose reduction/discontinuation of antipsychotic treatments on cognitive and social function in patients with FEP or schizophrenia. Therefore, the aim of this article is to provide an overview on which strategy is more beneficial for these outcomes.

## Materials and methods

### Search strategy and selection criteria

We searched PubMed, the Cochrane Library, and systematic reviews for randomized and non-randomized controlled trials published between 2007 and 2018, using the following search string; (schizophrenia OR psychosis OR first episode) AND (dose reduction OR discontinuation) AND (cognition OR social OR function OR relapse OR remission OR recovery). We also searched the reference lists of previous reviews, which compared the effects of medication maintenance vs. dose reduction/discontinuation on relapse, remission, cognition, and/or social function in FEP or schizophrenia (16–19). The searches were limited to English language articles and titles/abstracts. Due to the limited number of RCTs on functional outcome between the two groups (dose reduction/discontinuation vs. maintenance) in FEP, we included open-label randomized controlled trials and non-randomized prospective studies.

## Results

### Study design

We have identified five open-label and one double-blind studies comparing cognitive and/or social functional consequences between maintenance and dose reduction/discontinuation of antipsychotic treatments in patients with FEP or schizophrenia. Out of them, five were RCTs (3, 4, 20–23), whereas one was a non-randomized, prospective study (24). A summary of these studies, including the methodology and results, is presented in Table 1 (for comparisons between studies, relapse rates were calculated, where necessary, by diving the number of patients who relapsed during observation periods by the total number of patients in the corresponding group). Subjects included patients with schizophrenia, schizophrenia spectrum disorders, or FEP, with various regimens in terms of medication dose reduction/discontinuation, follow-up period, and outcome measures. Sample sizes ranged from 42 to 178, and the length of follow-up ranged from 28 weeks to 10-years.

**Table 1:** Cognition and social function in maintenance vs. dose reduction/discontinuation of antipsychotic drugs in psychosis.

**Study and year**	**No. of subjects**	**Subject population**	**Study design**	**Follow-up period**	**Outcome measure**	**Relapse rate (%)**	**Cognition**	**Social function**
Gaebel et al. (4)	MM 23/MD 21	Clinically stable first-episode schizophrenia (ICD-10), aged 18–56, with antipsychotic treatment for 12 months and 8 weeks	RCT, OL, during the last 1-year of the 2-years and 8 weeks follow-up, comparison MM with MD (targeted intermittent treatment after stepwise drug discontinuation), risperidone or haloperidol	1-year	GAF	MM 0 < MD 19^a^		GAF score (mean) MM (79.4) > MD (62.1)^a^ LQLP (mean) MM 5.3: MD 4.8, NS
Faber et al. (20)	MM 20/MD 22	FEP, having reached remission within 6 months of the start of second generation antipsychotics	RCT, OL, comparison MM with MD, risperidone, haloperidol or quetiapine	2–3 months	Stroop test, CPT, digit span, CVLT, TMT, verbal fluency, Symbol Substitution, Finger tapping		Improvement on Symbol Substitution, verbal fluency, TMT B MM < MD^a^	
Wunderink et al. (21)	MM 52/MD 51	FEP in remission for 6 months	RCT, OL, during the last 2-years of the follow-up, comparison MM with MD, doses below 1 mg of haloperidol equivalents	7-years	GSDS	MM 68.6:MD 61.5, NS *Symptomatic remission; MM 66.7: MD 69.2, NS*		% functional remission MM (19.6) < MD (46.2)^a^
Takeuchi et al. (22)	MM 30/MD 31	Schizophrenia (DSM-IV) in remission with respect to positive symptoms and treated with stable doses of olanzapine or risperidone	RCT, OL, comparison MM with MD (dose reduced by 50%), olanzapine or risperidone	28 weeks	RBANS	MM 3.0: MD 3.0, NS	Improvement in RBANS scale score (mean) MM (−0.1) < MD (+7.0)^a^	
Mayoral-van Son et al. (24)	MM 22/MD 46	FEP with antipsychotic treatment ≥ 18 months, stabilized at the lowest effective dose ≥ 3 months, clinical remission ≥ 12 months, functional recovery ≥ 6 months	Nonrandomized, prospective study, OL, comparison MM with MD (discontinuation), amisulpride, aripiprazole, haloperidol, olanzapine, quetiapine, risperidone, ziprasidone	3-years	DAS	MM 31.8 < MD 67.4^a^		% functional recovery (DAS global score = 0) MM 90.9: MD 84.8, NS
Chen et al. (3) and Hui et al. (23)	MM 89/MD 89	First-episode schizophrenia (DSM-IV) with full positive symptom resolution after about 2-years of antipsychotic treatment, aged 18–65	RCT, DB, comparison MM with MD (early discontinuation) for 12 months, quetiapine	10-years	SOFAS, RFS, SF-36	Relapse at 12 months MM 30 < MD 63^a^ Composite poor outcome at 10-years MM 21 < MD 39^a^		SOFAS score (mean) MM 61.9: MD 64.0, NS RFS total score (mean) MM 21.3: MD 21.5, NS SF-36: MCS score (mean) MM 50.2: MD 51.0, NS SF-36: PCS score (mean) MM 56.6: MD 56.9, NS

*CPT, continuous performance test; CVLT, California Verbal Learning Test; DAS, Disability Assessment Schedule; DB, double blind; FEP, first episode psychosis; GAF, Global Assessment of Functioning; GSDS, Groningen Social Disability Schedule; LQLP, Lancashire Quality of Life Profile; MD, medication dose reduction/discontinuation; MM, medication maintenance; OL, open label; RBANS, Repeatable Battery for the Assessment of Neuropsychological Status; RCT, randomized controlled trial; RFS, the Role Functioning Scale; SF-36, the 36-Item Short Form Health Survey; MCS, mental component summary; PCS, physical component summary; SOFAS, the Social and Occupational Functioning Assessment Scale; TMT, Trail-Making Test*.

a *Statistically significant; NS, no significant difference*.

### Outcome measures

Two studies included measures of cognition, while 4 studies used measures of social function at follow-up.

Faber et al. (20) used a test battery consisting of the Stroop 2 (color naming) and 3 (color-word naming) tests, continuous performance test (CPT), digit span forward and backward, California Verbal Learning Test, Trail-Making A and B, verbal (category) fluency (animals and professions), Symbol Substitution Test, and Finger tapping (25, 26). These tests have been shown to represent attention, working memory, verbal memory, cognitive speed of processing, and motor speed.

Takeuchi et al. (22) assessed cognition with the Repeatable Battery for the Assessment of Neuropsychological Status (RBANS) (27, 28). This battery yields scaled scores for 5 cognitive domains, i.e., immediate memory, visuospatial/constructional ability, language, attention, and delayed memory.

Gaebel et al. (4) assessed social function with the Global Assessment of Functioning (GAF) (29). This scale measures symptom severity, as well as psychological, social, and occupational functioning during specified periods, on a continuum from mental health (score 100) to mental illness (score 0) (30). QOL was measured by the Lancashire Quality of Life Profile (LQLP) (31). The LQLP focuses on nine specific domains; living situation, family, social relationships, leisure activities, work/education, finances, personal safety, health, and religion. The questions pertaining to the subjective QOL appraisal allow patients to rate their satisfaction on a seven-point scale (32).

Wunderink et al. (21) evaluated social function with the Groningen Social Disability Schedule (GSDS) (33), a semi-structured investigator-based interview measuring disabilities in social function. Seven items from the GSDS were used; self-care, housekeeping, family relationship, partner relationship, relationship with peers, community integration, and vocational functioning. A patient with functional remission should function adequately in all 7 domains with none or only a minimal disability in all of them (not allowing a score of 2 or 3).

Mayoral-van Son et al. (24) used the global disability item from the Spanish version of the Disability Assessment Schedule (DAS). This item has a score range from 0 (no disability) to 5 (gross disability). They dichotomized functional status into “functional recovery” and “functional deficits.” The “functional recovery” status indicates that the patient is currently participating in part-time (paid and fewer than 35 h per week) or full-time work or study with the same or better level of performance as before the psychotic episode, and has no functional disability (score of 0 in the DAS).

Hui et al. (23) assessed social function with the Social and Occupational Functioning Assessment Scale (SOFAS) and the Role Functioning Scale (RFS), as well as health-related QOL with the 36-Item Short Form Health Survey (SF-36). The SOFAS assesses social and occupational functioning, whose scores are not directly influenced by overall severity of the individual's psychological symptoms (34, 35). The scores range from 1 to 100, with lower scores representing impaired functioning. The RFS measures the functioning level in patients with psychiatric disorders, focusing on four domains; working productivity, independent living and self-care, immediate social network relationships, and extended social network relationships (36). Each domain is rated on a 7-point scale, with lower scores representing lower functioning. The SF-36 consists of 36 questions on functional health and well-being (37), as summarized by two indices, i.e., the mental component summary (MCS) and physical component summary (PCS). The MCS includes four domains; vitality (energetic or fatigued), social functioning, role limitations because of emotional problems, and general mental health (psychological distress and well-being), while the PCS consists of four domains; physical functioning, role limitations because of physical health problems, bodily pain, and general health perceptions. SF-36 scores range from 0 to 100, with higher scores indicating better functional health and well-being.

### Relapse rate

In the reviewed studies, relapse rates range from 3.0 to 67.4% in dose reduction/discontinuation groups, and 0–68.6% in medication maintenance groups. Three trials (3, 4, 24) with a follow-up period less than 3-years reported a higher rate of relapse in the dose reduction/discontinuation groups, while a 7-year follow-up study (21) found that relapse rates of the two groups (dose reduction vs. maintenance) were equal. In a 10-year follow-up study (23), the incidence of persistent positive symptoms, requirement for clozapine, or death by suicide occurred in 39% in the discontinuation group and 21% in the maintenance group (risk ratio 1.84, 95% CI 1.15–2.96; *p* = 0.01).

### Cognition

Faber et al. (20) found that the medication discontinuation group showed a significantly greater improvement than the maintenance group on scores of the Symbol Substitution Test (*F* = 4.49, *df* = 1.40, *P* < 0.05), verbal fluency task (*F* = 6.11, *df* = 1.40, *p* < 0.05) and Trail-Making Test-B (*F* = 5.54, *df* = 1.40, *p* < 0.05) at 2–3 months after the start of dose reduction/discontinuation. Similarly, Takeuchi et al. (22) reported that the dose reduction group showed a significantly greater improvement in RBANS scores compared with the maintenance group [mean (SD), 7.0 (7.1) vs. −0.1 (8.0), *p* < 0.001].

### Social function

Gaebel et al. (4) reported that the medication maintenance group showed a greater mean GAF score at 1-year follow-up point than that in the dose reduction/discontinuation group [79.4 (10.1) vs. 62.1 (16.7), *p* < 0.001]. On the other hand, no between-group difference was found for QOL [5.3 (1.2) vs. 4.8 (0.9), NS]. By contrast, Wunderink et al. (21) found that the functional remission rate after 7-years was significantly higher for the dose reduction/discontinuation group compared with the maintenance group (19.6 vs. 46.2%, *p* < 0.01). On the other hand, Mayoral-van Son et al. (24) did not find a difference in the functional status at the 3-year follow-up point between the two groups (90.9 vs. 84.8%, NS). Likewise, Hui et al. (23) did not observe a significant difference in the functional status at the 10-year follow-up point between the two groups; [61.9 (9.6) vs. 64.0 (8.9), NS] on SOFAS [21.3 (3.4) vs. 21.5 (2.8), NS] on RFS Total, [50.2 (9.1) vs. 51.0 (8.4), NS] on SF-36 (MCS) and [56.6 (7.6) vs. 56.9 (6.6), NS] on SF-36 (PCS).

## Discussion

The present review identified 2 studies reporting on cognition and 4 studies on social function, which compared functional outcomes between medication maintenance vs. dose reduction/discontinuation patients with FEP or schizophrenia. In spite of abundant information on relapse rates, only the limited number of studies have dealt with these outcomes, particularly, cognition. Of note, all studies except one (4) reported the advantage of the dose reduction/discontinuation method over the maintenance strategy in terms of functional outcomes.

In terms of social function, a trial with a 1-year follow-up period (4) found a greater improvement in the maintenance group, while there was no significant group difference 3-years and 10-years after the start of follow-up (23, 24). On the other hand, the study with a 7-year follow-up period (21) observed a superiority for the dose reduction/discontinuation regimen. The difference in study design may account for the discrepant results. For example, social function was measured by the GAF in the 1-year follow-up study (4). The GAF assesses psychotic symptoms and overall functional ability simultaneously; when symptom severity and level of functioning are discordant, clinicians are directed to use the rating that reflects the lower of the two levels (30, 38). In the 1-year follow-up study (4), the high relapse rate in the dose reduction/discontinuation group may have affected symptom severity, leading to worsening of GAF scores (assumed to represent “function” status). The use of standardized scale to assess real-world social function, independent of symptom severity, would be desired to circumvent this issue. Regarding the study with a 7-year follow-up period (2, 21), both groups were similar in their interventions, because about 80% in the dose reduction/discontinuation group *failed to discontinue* drug treatment (39). In addition, follow-up was generally naturalistic and unblinded, so there could be a difference in unmeasured psychosocial aspects, such as community care and number of visits (39). Furthermore, the differences in diagnostic categories in the 2 groups may be a plausible explanation toward the significantly better recovery and functional remission in the dose reduction/discontinuation group (40). Further study is warranted to determine the effect of length of observation periods on social function in patients who (dis)continue medications.

A greater improvement in cognition for the dose reduction/discontinuation group was reported in two studies (20, 22) with a relatively short follow-up period of less than 3 months. Fu et al. (11) found that performance on tests of attention and verbal working memory predicted social function, as measured by the Global Functioning (Social and Role) (11), in addition to temporal associations between social function vs. attention, verbal working memory, and verbal learning memory at baseline. Also, a longer observation period revealed that a greater cumulative lifetime antipsychotic use led to poorer cognitive performance in later life in patients with schizophrenia, which may be caused by disorganization symptoms (41). Future studies should consider type of cognitive domains and potential effects of key clinical features on cognitive and functional outcomes.

Part of the results from this review suggests that dose reduction/discontinuation of antipsychotic treatments after remission leads to better functional outcome in FEP and schizophrenia. In addition, Wunderink et al. (21) reported that milder negative symptoms, living together, and better social function at baseline are associated with better functional outcomes. Meanwhile, we often experience chronic patients exhibiting severe functional impairment as a result of repeated relapses after discontinuation of antipsychotic drugs. In fact, Mayoral-van Son et al. (24) found that relapsed patients showed more severe symptoms and poorer functional status at the end of follow-up periods. Further, Gaebel et al. (42) found that social function was significantly poorer in patients who relapsed after drug discontinuation compared to those without relapse. These observations indicate a need for the search of predicting factors to identify patients who need continued medications.

Previous studies have attempted to identify patients who will be benefitted or jeopardized by dose reduction/discontinuation of antipsychotic drugs. Accordingly, Wunderink et al. (21) found that a short duration of untreated psychosis was the strongly associated with achievements of symptom remission. Moreover, Alvarez-Jimenez et al. (16) noted that risk factors of relapse after discontinuation or dose reduction include diagnosis of schizophrenia, longer duration of illness, and poor pre-morbid functioning. They also reported that psychosocial interventions for FEP coupled with antipsychotic drugs were effective in preventing relapses (43). Furthermore, the guidelines devised by the International Early Psychosis Association (1) recommend that the minimal dose antipsychotic medication should be continued for preventing relapse and impairment of functions in FEP patients with risks of relapse.

In conclusion, although dose reduction/discontinuation of antipsychotic medication may be associated with higher relapse rates, this strategy may improve cognitive outcomes in some patients with FEP or schizophrenia. In this line, predictors for successful dose reduction/discontinuation deserve further explorations. So far, information on cognition and social function is relatively sparse. These measures of functional outcome should be considered in deciding which strategy of antipsychotic treatments is encouraged in individual cases.

## Author contributions

TS planned and initiated this work. YO performed the literature search and drafted the first manuscript. Both authors revised the manuscript and approved the final version.

### Conflict of interest statement

The authors declare that the research was conducted in the absence of any commercial or financial relationships that could be construed as a potential conflict of interest.
